# Supplementary material for: Base editing in human cells with monomeric DddA-TALE fusion deaminases
Source: Nat Commun. 2022 Jul 12;13:4038. doi: 10.1038/s41467-022-31745-y (PMC9276701; doi:10.1038/s41467-022-31745-y)

Supplementary Information

Base editing in human cells with monomeric DddA-TALE fusion deaminases

Young Geun Mok[1, 3], Ji Min Lee[1, 2, 3], Eugene Chung[1, 2, 3], Jaesuk Lee[1, 2], Kayeong Lim[1], Sung-Ik Cho[1, 2] and Jin-Soo Kim[1]

[1] Center for Genome Engineering, Institute for Basic Science, Daejeon 34126, Republic of Korea. [2] Department of Chemistry, Seoul National University, Seoul, Republic of Korea.

[3] These authors contributed equally to this work.

Correspondence should be addressed to J.-S.K. (jskim01@snu.ac.kr).

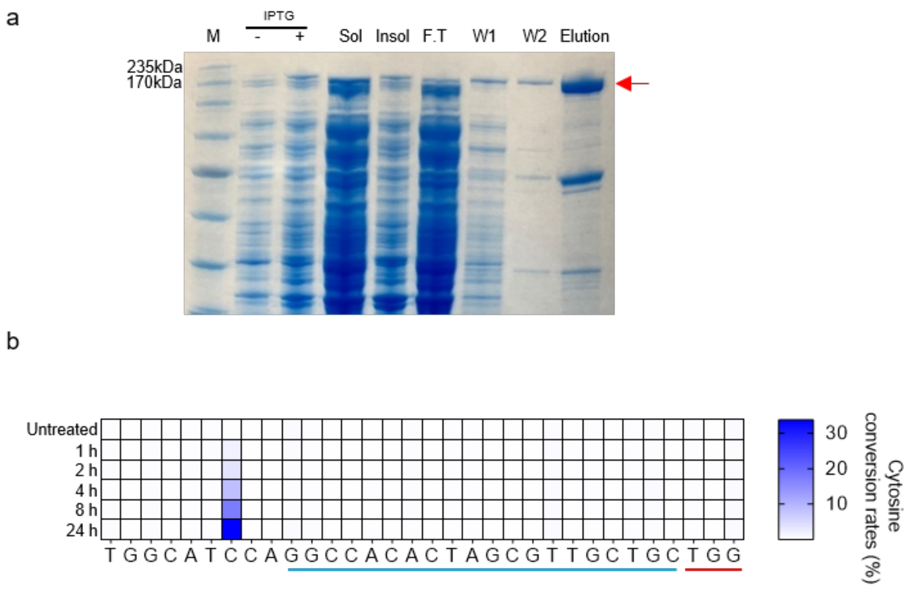

Supplementary Figure 1. *In vitro* assay to measure the deaminase activity of E1347A-D10A nCas9. **a**, Nickel agarose bead-based purification of His-tagged E1347A-D10A nCas9 protein from *E. coli* cell lysates was monitored using polyacrylamide gel electrophoresis. Coomassie blue was used to stain the gel. Lane 1, molecular weight indicators, with sizes of representative markers indicated to the left. Lane 2, sample from cells in which protein expression was not triggered by IPTG. Lane 3, Sample from cells in which protein expression was stimulated with IPTG. Lane 4, soluble fraction after sonication. Lane 5, insoluble fraction after sonication. Lane 6, flow-through fraction from the column. Lane 7, first wash fraction. Lane 8, second wash fraction. Lane 9, elution fraction. The red arrow indicates the E1347A-D10A nCas9 protein. **b**, Time-dependent *in vitro* activity of E1347A-D10A nCas9 measured by targeted deep sequencing of a PCR amplicon containing the *TYRO3* target. The protospacer and PAM are underlined in blue and red, respectively. The colors in the heat map were determined from three independent experiments.

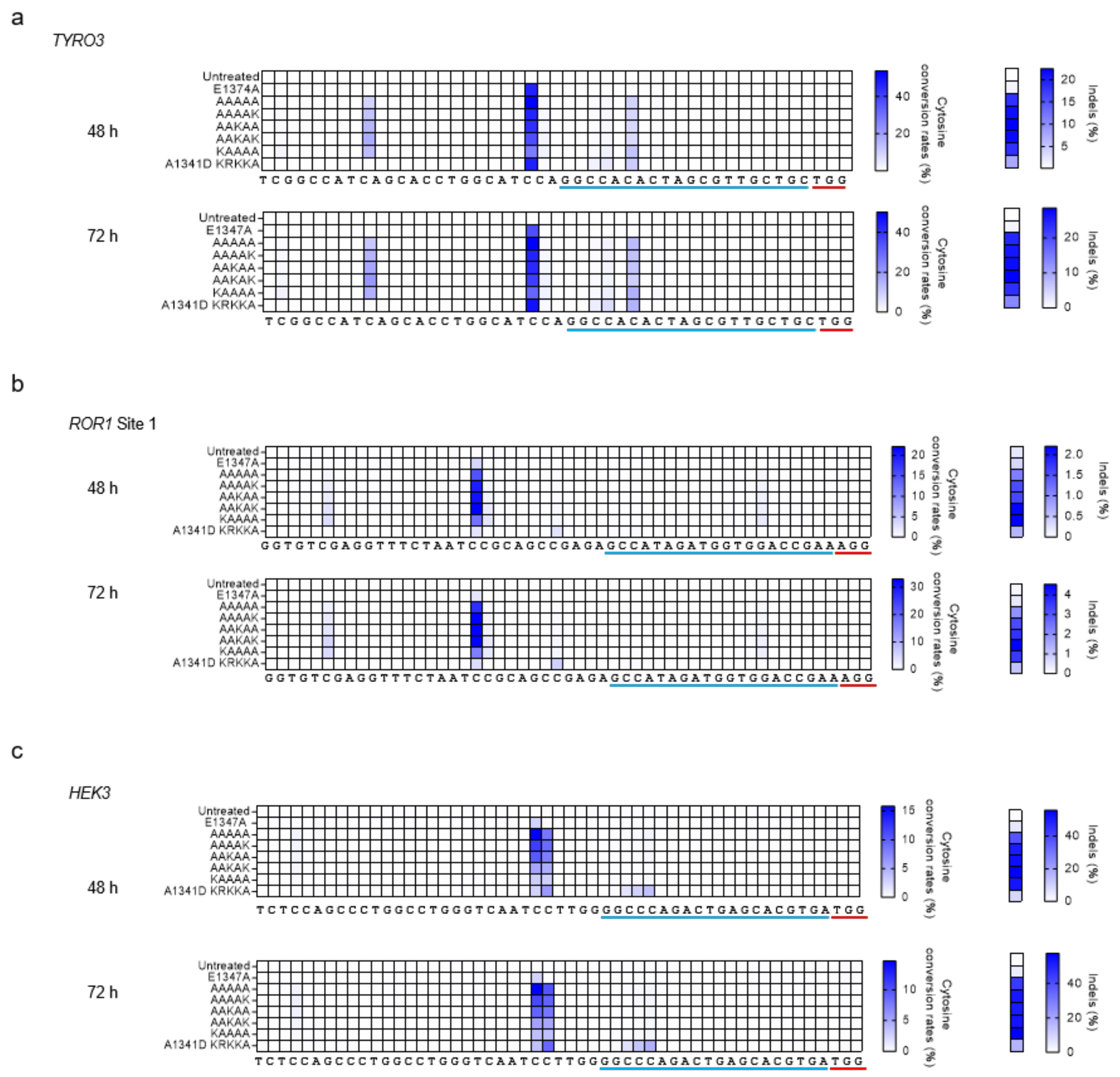

Supplementary Figure 2. Heat maps showing the C-to-T conversion frequencies frequencies induced in HEK293T cells by Ala-substituted DddA$_{tox}$ variants fused to the N terminus of D10A nCas9 at various positions in the *TYRO3* site (a), *ROR1* site 1 (b), and a *HEK3* site (c) 48 and 72 h after transfection. Indel frequencies are indicated to the right. The cytosine conversion rates and indel frequencies were measured by targeted deep sequencing. The protospacer and PAM are underlined in blue and red, respectively. Colors in the heat maps were determined from three independent experiments.

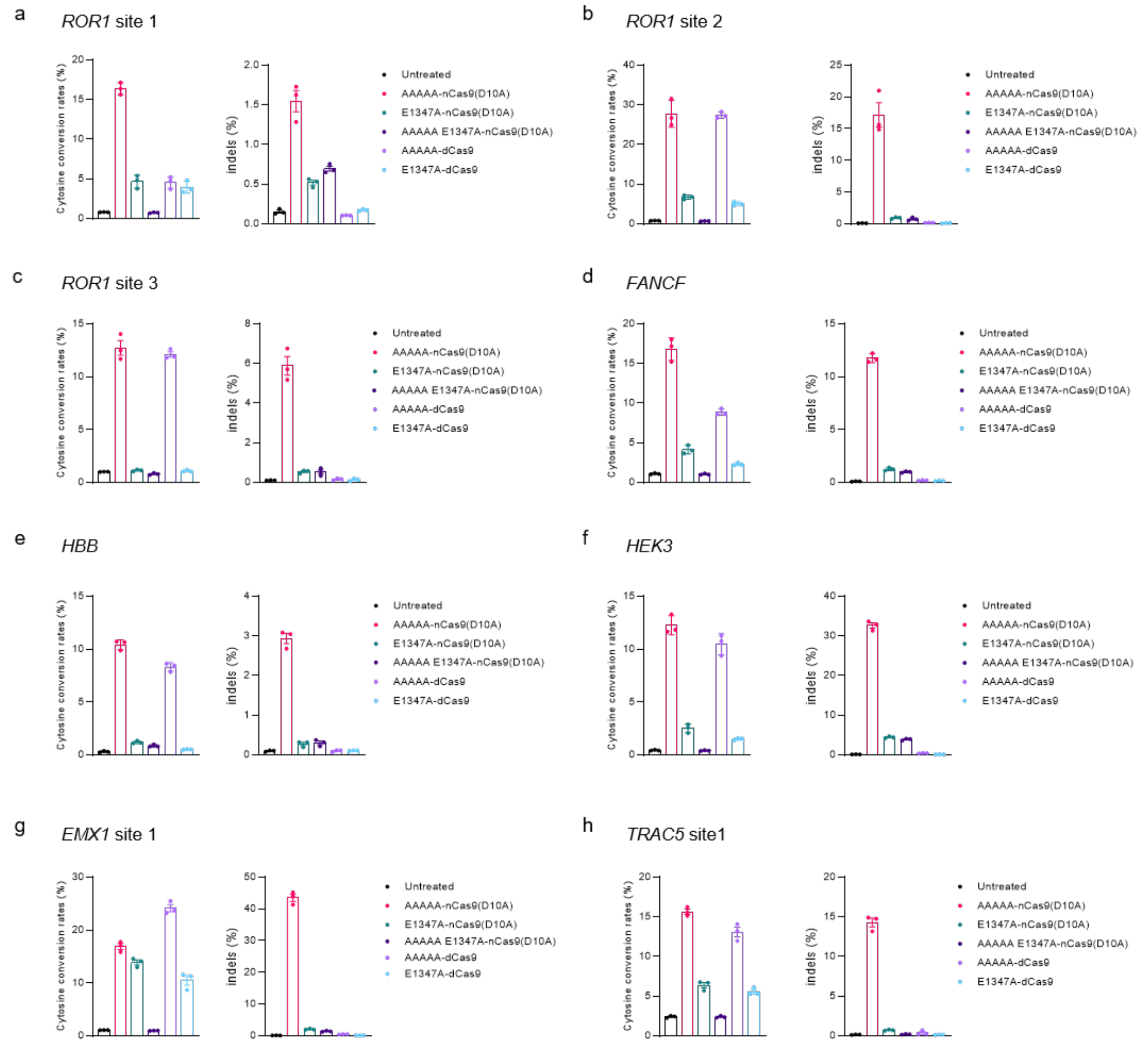

Supplementary Figure 3. Editing and indel frequencies induced in HEK293T cells by $DddA_{tox}$ AAAAA, E1347A, and AAAAA E1347A variants fused to the N terminus of D10A nCas9 or dCas9 at *ROR1* site 1 (a), *ROR1* site 2 (b), *ROR1* site 3 (c), a *FANCF* site (d), an *HBB* site (e), the *HEK3* site (f), *EMX1* site 1 (g), and *TRAC5* site 1. The cytosine conversion rates and indel frequencies were measured by targeted deep sequencing. Means ± s.e.m. were determined from three independent experiments.

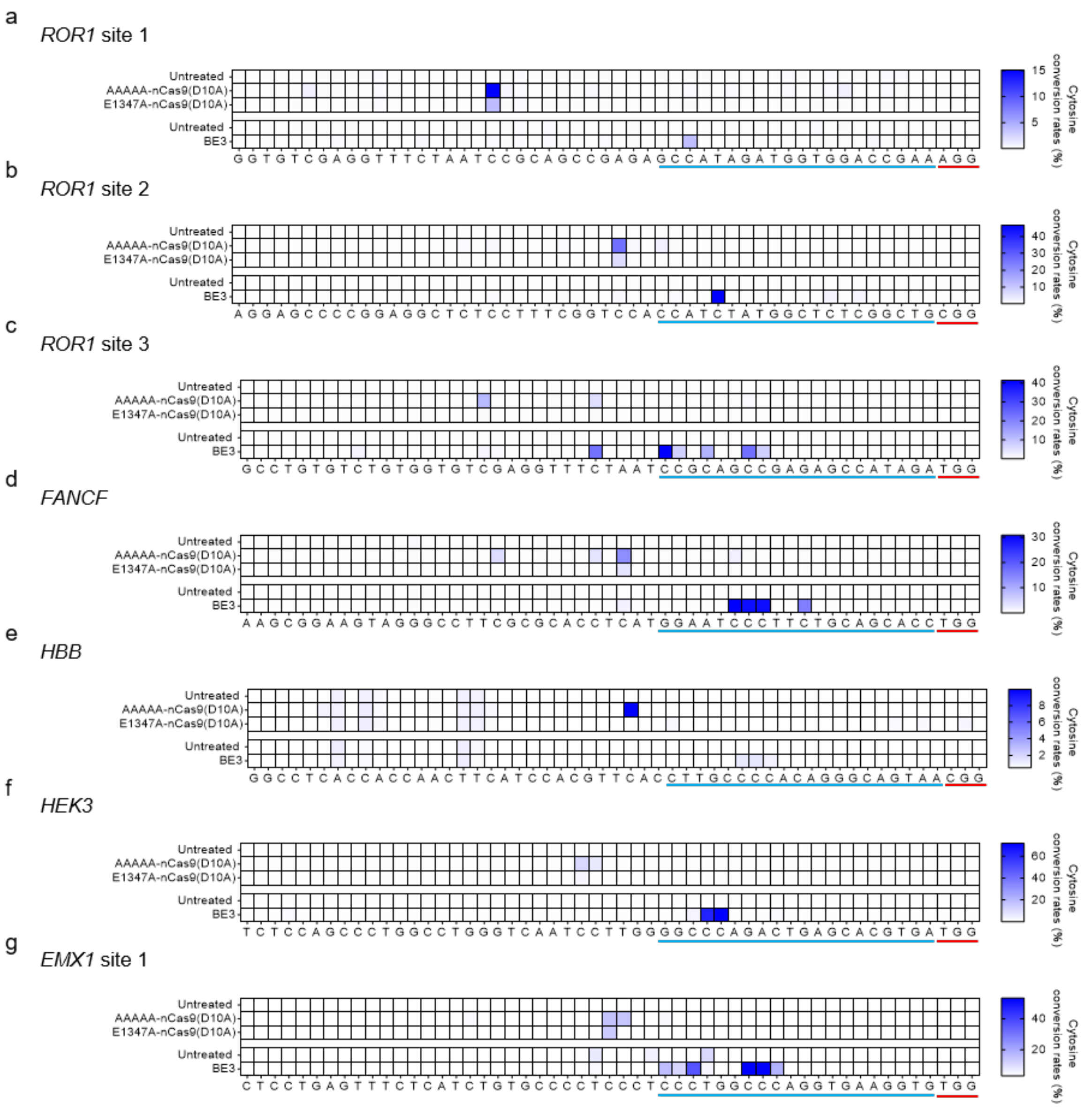

Supplementary Figure 4. Heat maps showing the C-to-T conversion frequencies induced in HEK293T cells by DddA$_{tox}$ AAAAA and E1347A fused to the N terminus of D10A nCas9, and by BE3, at various positions in *ROR1* site 1 (a), *ROR1* site 2 (b), *ROR1* site 3 (c), the *FANCF* site (d), the *HBB* site (e), the *HEK3* site (f), and *EMX1* site 1 (g). The protospacer and PAM are underlined in blue and red, respectively. The cytosine conversion rates frequencies were measured by targeted deep sequencing. Colors in the heat map were determined from three independent experiments.

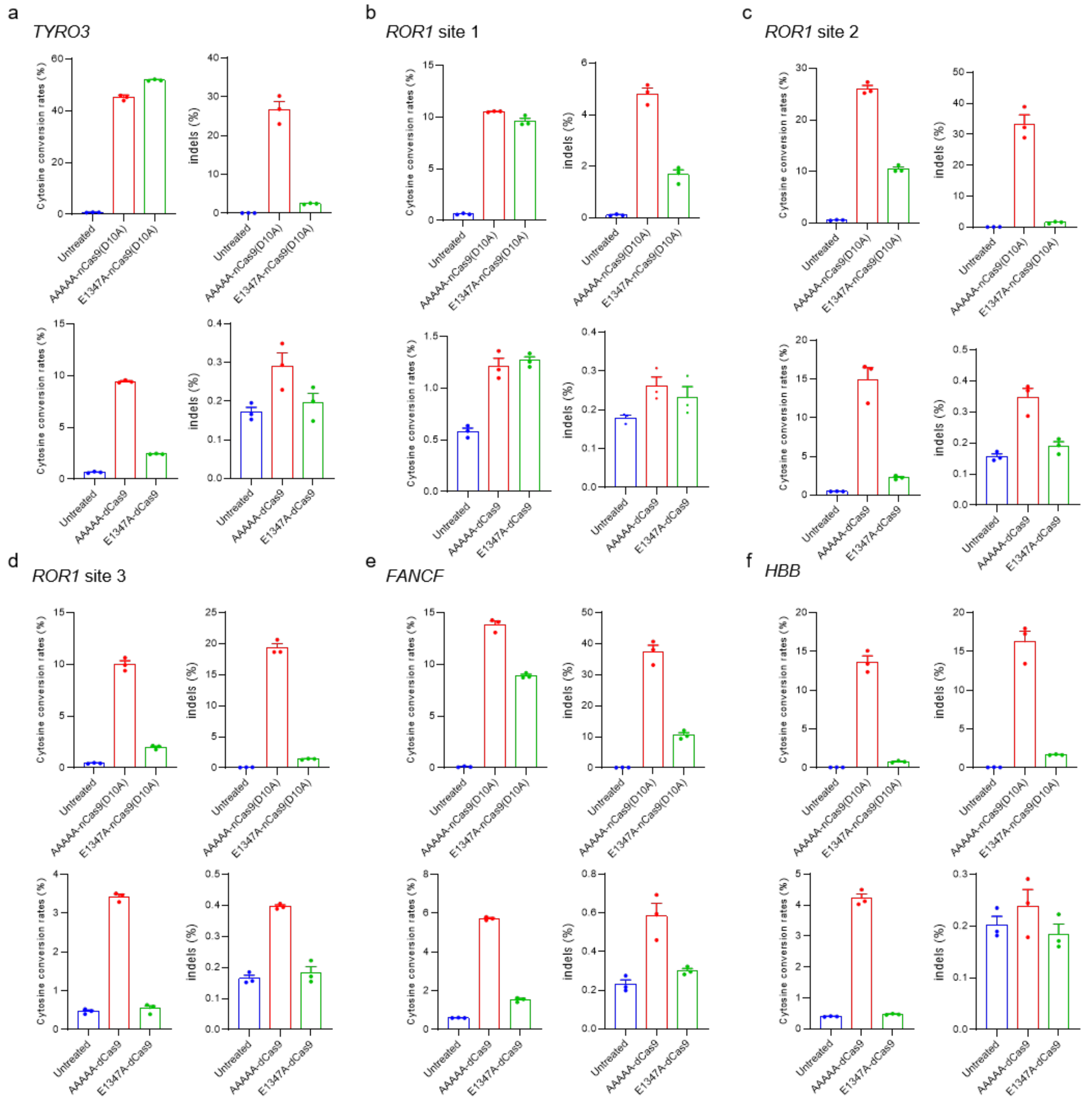

Supplementary Figure 5. Editing and indel frequencies induced in HeLa cells by DddA$_{tox}$ AAAAA and E1347A variants fused to the N terminus of D10A nCas9 or dCas9 at the *TYRO3* site (a), *ROR1* site 1 (b), *ROR1* site 2 (c), *ROR1* site 3 (d), the *FANCF* site (e), and the *HBB* site (f). The cytosine conversion rates and indel frequencies were measured by targeted deep sequencing. Means ± s.e.m. were determined from three independent experiments.

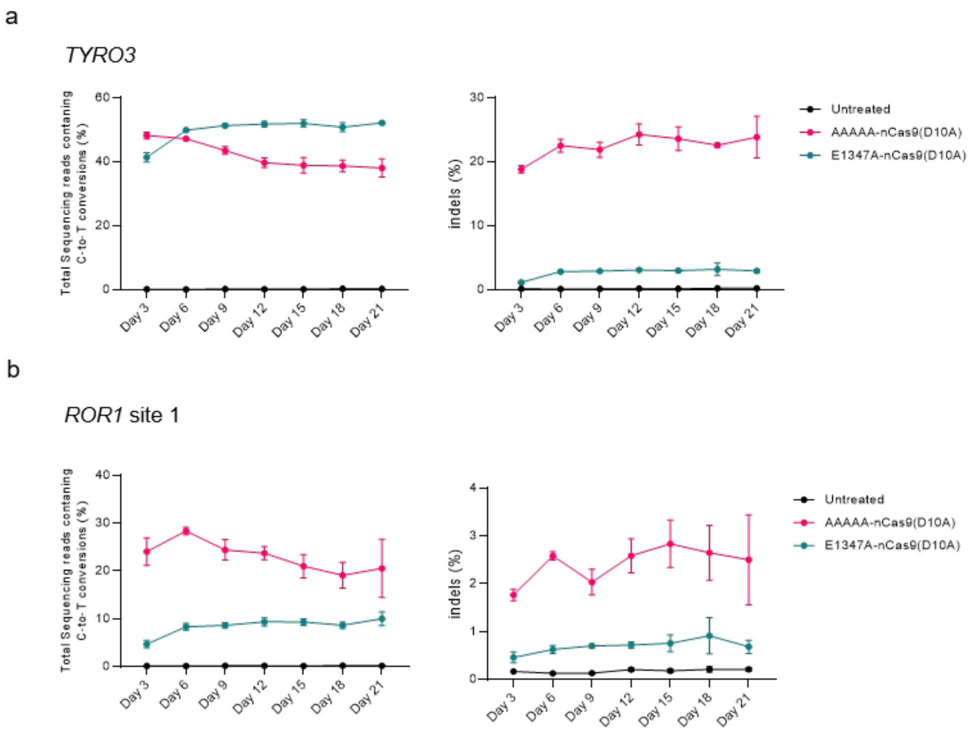

Supplementary Figure 6. Time-dependence of editing and indel frequencies induced in HEK293T cells by DddA$_{tox}$ AAAAA and E1347A variants fused to the N terminus of D10A nCas9 at the *TYRO3* site (position C$_{-3}$) (a) and *ROR1* site 1 (position C$_{-12}$) (b). The base editing and indel frequencies were measured by targeted deep sequencing. Means ± s.e.m. were determined from three independent experiments.

a AAAAA-D10A nCas9

TYRO3

	Editing frequency (%)		
	C-6	C3	C16
#1	0.13	0.23	0.08
#2	0.09	99.8	0.11
#3	0.12	99.6	47.1
#4	0.08	22.2	0.32
#5	0.05	40.8	38.9
#6	3.8	50.9	1.16
#7	51.5	99.7	47.5
#8	0.37	6.5	2.9
#9	0.1	0.18	0.12
#10	0.09	99.8	48.9
#11	50.8	99.7	0.07

ROR1 site *1*

	Editing frequency (%)	
	C12	C25
#1	57.8	0.11
#2	0.09	0.06
#3	0.1	0.1
#4	0.14	0.14
#5	57.4	0.09
#6	0.07	0.08
#7	4.1	0.09
#8	0.06	0.13
#9	1.7	0.12
#10	27.5	0.19
#11	0.1	0.1
#12	0.11	0.11
#13	0.12	0.09
#14	51	19.4
#15	77.1	22.9
#16	0.07	0.07
#17	60.5	0.06

b E1347A-D10A nCas9

TYRO3

	Editing frequency (%)	
	C3	C16
#1	0.16	0.08
#2	26.4	0.08
#3	0.1	0.09
#4	50.1	0.11
#5	0.15	0.13
#6	51.8	0.07
#7	90.5	0.08
#8	0.12	0.12
#9	60.6	0.08
#10	99.4	0.13
#11	50.4	0.1
#12	50.5	0.12
#13	99.5	0.12
#14	77.7	0.11
#15	49.5	0.12
#16	48.4	0.11
#17	30	0.06
#18	49.4	0.08
#19	0.11	0.15
#20	1.75	0.09
#21	50.2	0.09

ROR1 site *1*

	Editing frequency (%)	
	C12	C25
#1	0.06	0.12
#2	0.09	0.07
#3	1.11	0.15
#4	0.08	0.08
#5	0.09	0.13
#6	25.4	0.11
#7	0.07	0.15
#8	0.09	0.1
#9	0.06	0.09
#10	0.08	0.05
#11	0.08	0.09
#12	20.3	0.12
#13	0.04	0.13
#14	0.06	0.09
#15	20.9	0.12
#16	0.04	0.1
#17	0.06	0.1
#18	0.09	0.08
#19	0.09	0.08
#20	0.06	0.15
#21	0.05	0.11
#22	0.07	0.11
#23	21.1	0.11

Supplementary Figure 7. mtDNA editing frequencies in single cell-derived clones. Frequencies were obtained at the *TYRO3* site (a) and *ROR1* site 1 (b) after treatment with AAAAA-D10A nCas9 and E1347A-D10A nCas9, respectively, in HEK293T cells. The editing frequencies were measured by targeted deep sequencing.

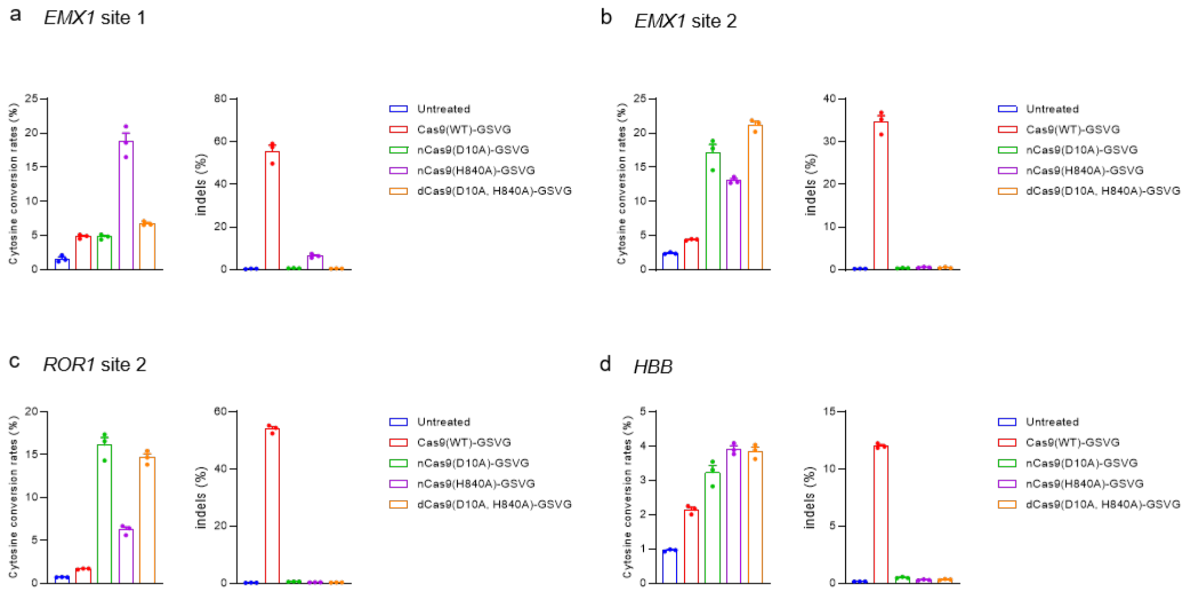

Supplementary Figure 8. Editing and indel frequencies induced in HEK293T cells by the DddA$_{tox}$ GSVG variant fused to the C terminus of Cas9, D10A nCas9, H840A nCas9, or dCas9 at *EMX1* site 1 (a), *EMX1* site 2 (b), *ROR1* site 2 (c), and the *HBB* site (d). The cytosine conversion rates and indel frequencies were measured by targeted deep sequencing. Means ± s.e.m. were determined from three independent experiments.

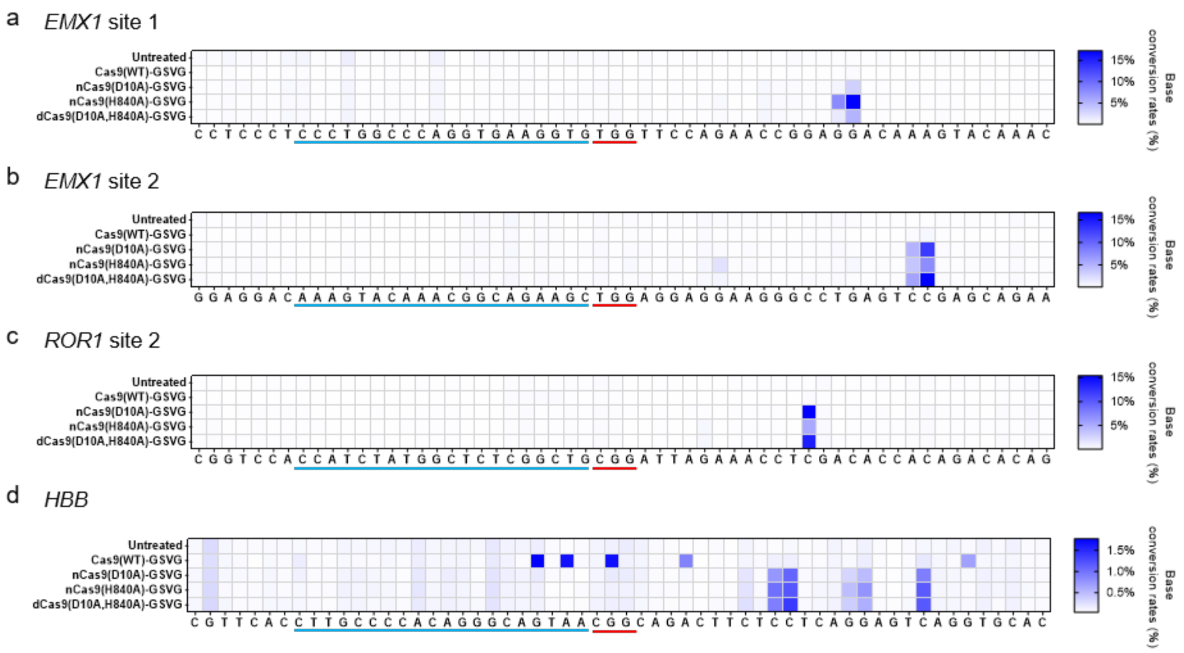

Supplementary Figure 9. Heat maps showing C-to-T and G-to-A conversion frequencies induced in HEK293T cells by the DddA$_{tox}$ GSVG variant fused to the C terminus of Cas9, D10A nCas9, H840A nCas9, or dCas9 at various positions in *EMX1* site 1 (a), *EMX1* site 2 (b), *ROR1* site 2 (c), and the *HBB* site (d). The base conversion rates were measured by targeted deep sequencing. The protospacer and PAM are underlined in blue and red, respectively. Colors in the heat map were determined from three independent experiments.

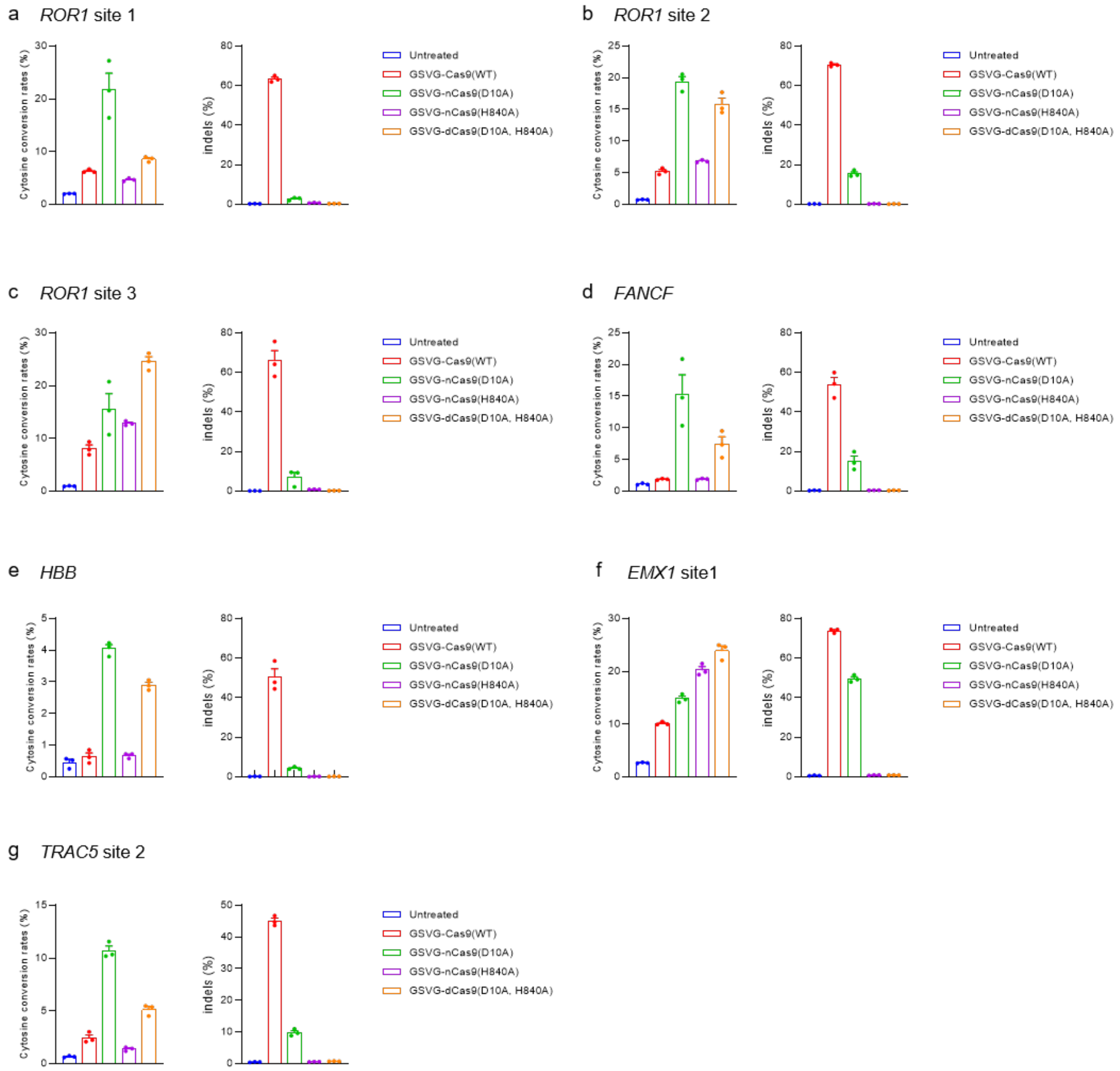

Supplementary Figure 10. Editing and indel frequencies induced in HEK293T cells by the DddA_{tox} GSVG variant fused to the N terminus of Cas9, D10A nCas9, H840A nCas9, or dCas9 at *ROR1* site 1 (a), *ROR1* site 2 (b), *ROR1* site 3 (c), the *FANCF* site (d), the *HBB* site (e), *EMX1* site 1 (f), and *TRAC5* site 2. The cytosine conversion rates and indel frequencies were measured by targeted deep sequencing. Means ± s.e.m. were determined from three independent experiments.

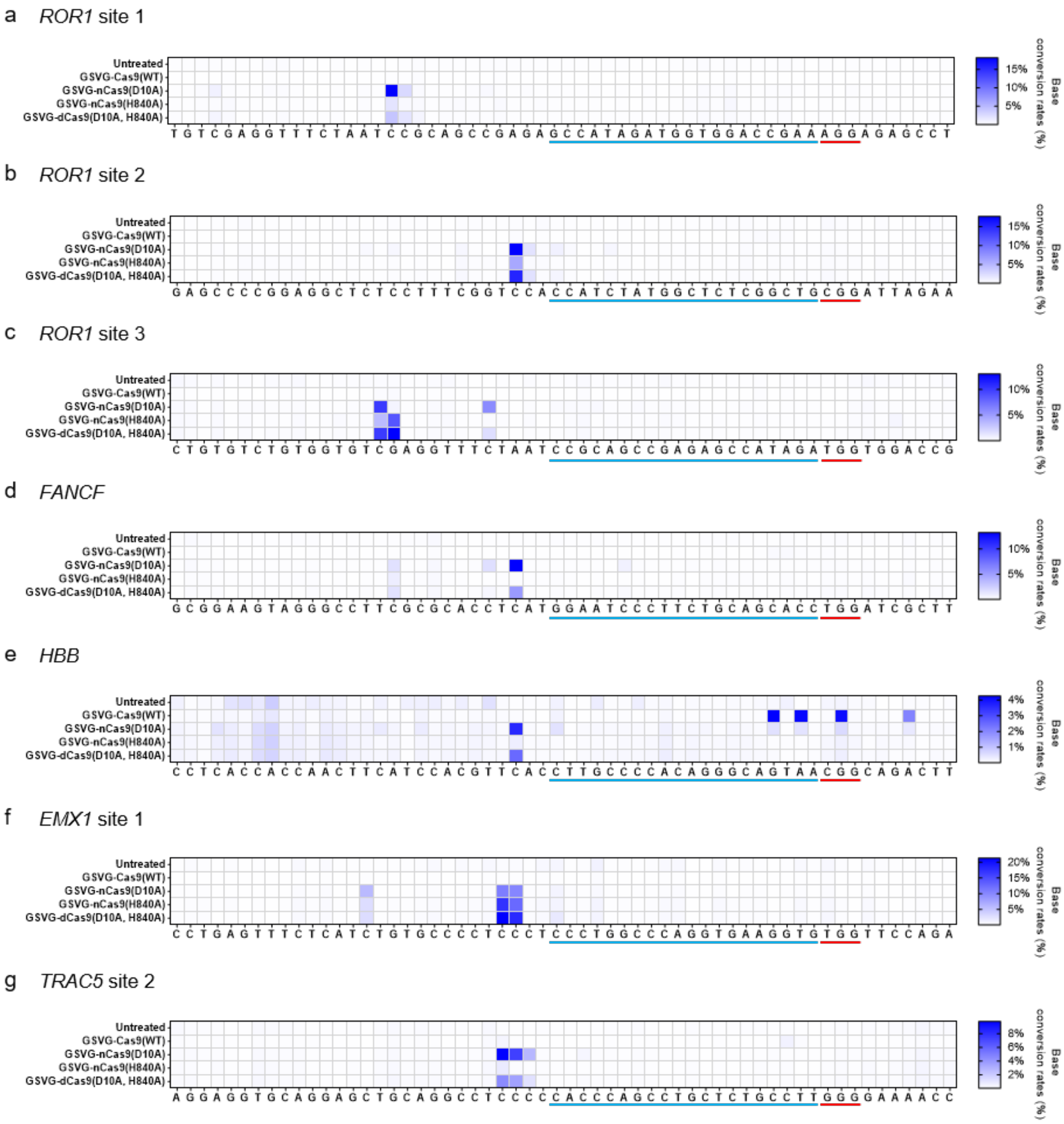

Supplementary Figure 11. Heat maps showing C-to-T and G-to-A conversion frequencies induced in HEK293T cells by the DddA$_{tox}$ GSVG variant fused to the N terminus of Cas9, D10A nCas9, H840A nCas9, or dCas9 at *ROR1* site 1 (a), *ROR1* site 2 (b), *ROR1* site 3 (c), the *FANCF* site (d), the *HBB* site (e), *EMX1* site 1 (f), and *TRAC5* site 2. The base conversion rates were measured by targeted deep sequencing. The protospacer and PAM are underlined in blue and red, respectively. Colors in the heat maps were determined from three independent experiments.

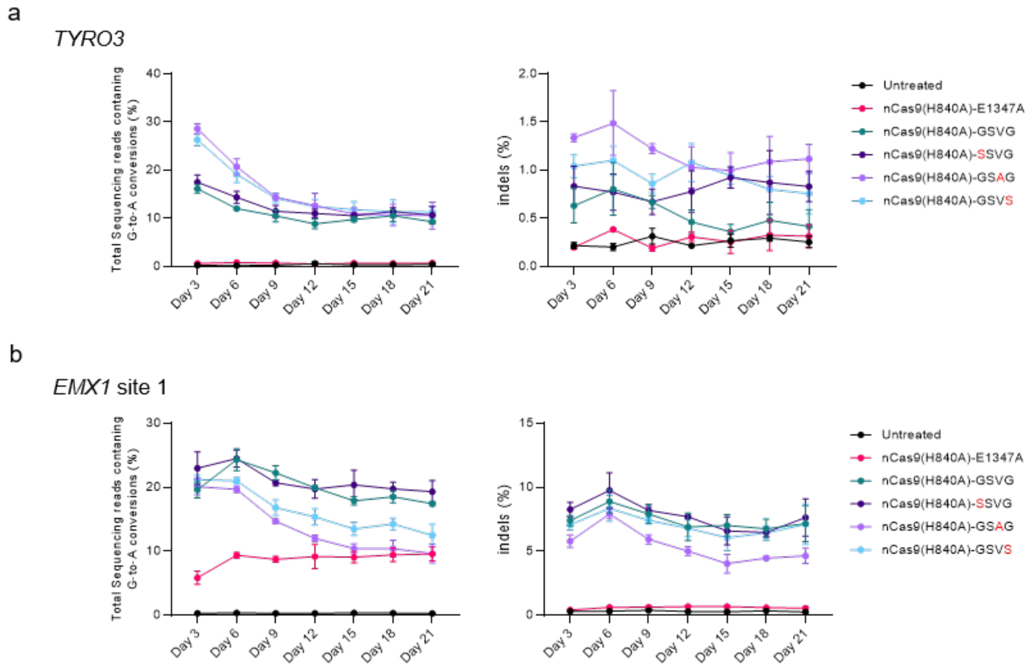

Supplementary Figure 12. Time-dependence of editing and indel frequencies induced in HEK293T cells by DddA$_{tox}$ E1347A, GSVG, SSVG, GSAG, and GSVS variants fused to the C terminus of H840A nCas9 at the *TYRO3* site (position G_{40}) (a) and *EMX1* site 2 (position G_{38}) (b). The guanine conversion rates and indel frequencies were measured by targeted deep sequencing. ± s.e.m. were determined from three independent experiments.

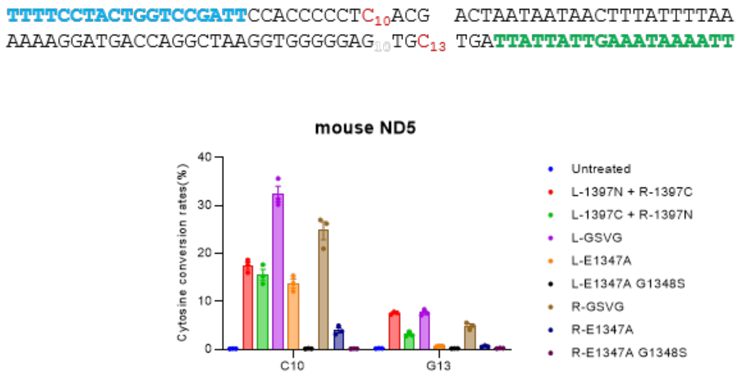

Supplementary Figure 13. Mitochondrial base editing frequencies induced by the indicated DdCBEs and mDdCBEs in mouse NIH3T3 cells at the *ND5* site. Target cytosines are shown in red and the left and right TALE-binding sites are shown in blue and green, respectively. The cytosine conversion rates were measured by targeted deep sequencing. Means ± s.e.m. were determined from three independent experiments.

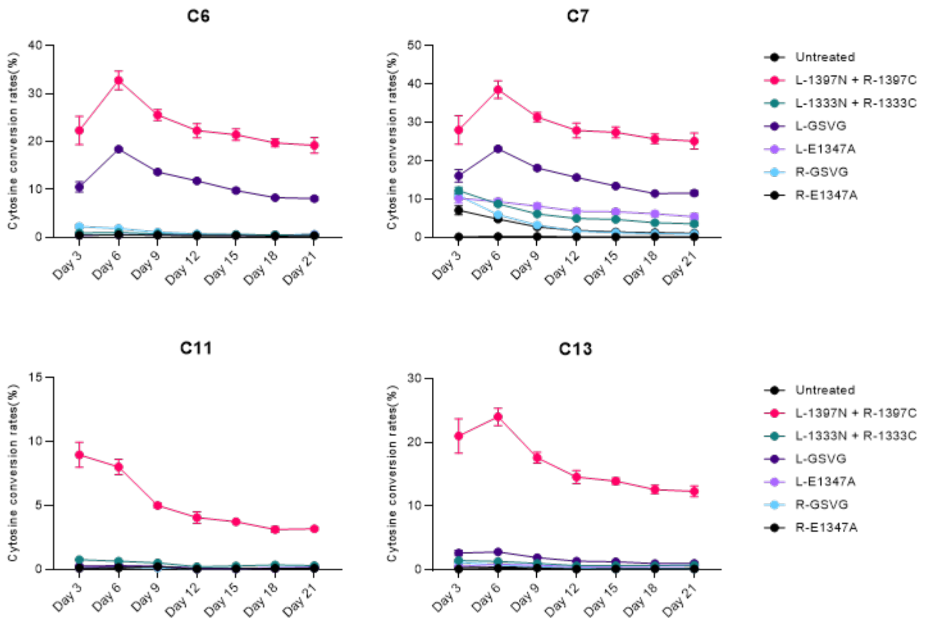

Supplementary Figure 14. Time-dependence of editing frequencies induced by the indicated DdCBEs and mDdCBEs in HEK293T cells. Editing efficiencies at each target cytosine in the *ND6* site are shown. The cytosine conversion rates were measured by targeted deep sequencing. Means ± s.e.m. were determined from three independent experiments.

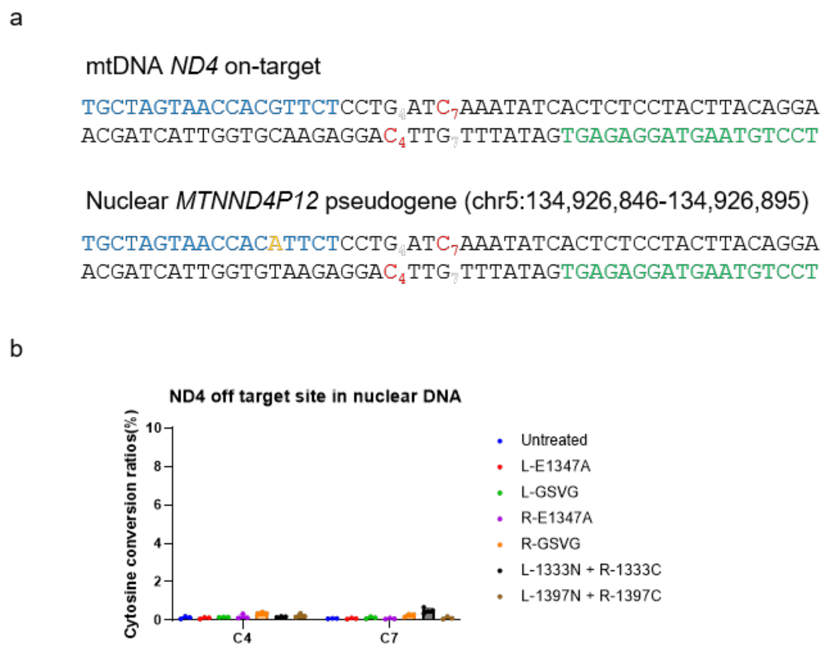

Supplementary Figure 15. Off-target editing activity of mitochondrially-targeted DdCBEs and mDdCBEs in the nuclear DNA of HEK293T cells. **a,** The on-target editing site in the mitochondrial *ND4* gene and the corresponding nuclear DNA sequence with the highest homology. The nucleotide mismatch between the mtDNA and the nuclear *MTNND4P12* pseudogene is shown in yellow. **b,** Editing frequencies induced by the indicated DdCBEs and mDdCBEs at the off-target site in *MTNND4P12*. Target cytosines are shown in red and the left and right TALE-binding sites are shown blue and green, respectively. The cytosine conversion rates were measured by targeted deep sequencing. Means ± s.e.m. were determined from three independent experiments.

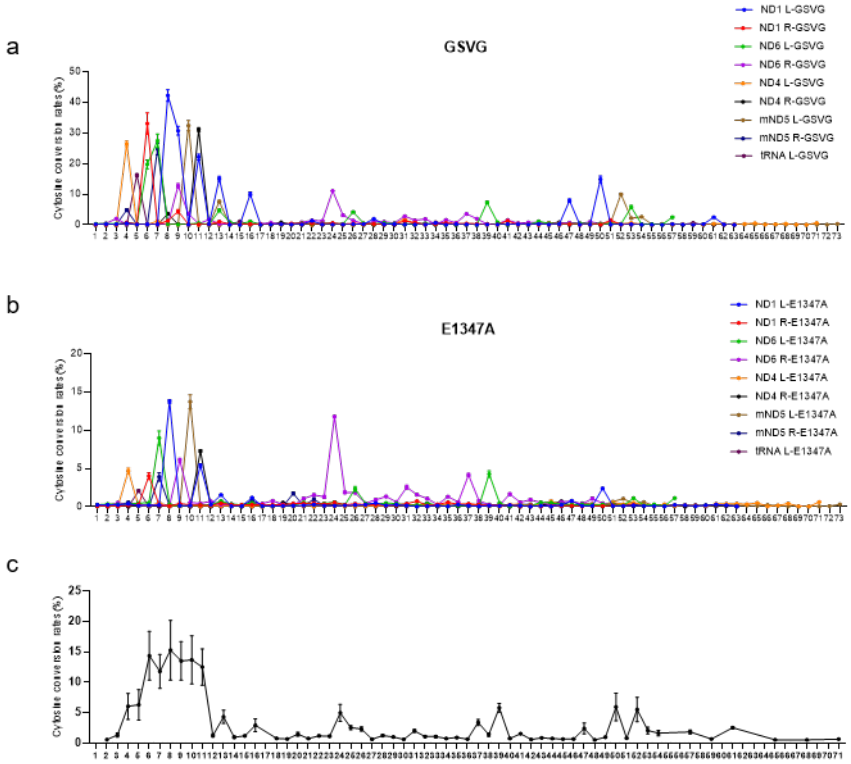

Supplementary Figure 16. Defining the mDdCBE editing window. Editing frequencies at each nucleotide position downstream of the TALE-binding sequence were obtained at 9 sites following treatment with mDdCBEs containing either the GSVG variant (a) or the E1347A variant (b). Summary of the 18 sets of results (c). Means ± s.e.m. were determined from three independent experiments.

Supplementary Table 1.

Name	Sequence 5` to 3`
DddA F	cccaagcttgccaccatgggatccggcagctacgccctgggtccgtatcag
DddA R	cccgggagtctcgctgccgctgcaaccgcctttggtcgggctcttcggg
K1402A R	cccgggagtctcgctgccgctgcaaccgcctttggtcgggctcttcgggctgttgctgtt accggtaaacactttggtttcaccggtagcgccgcgCGCcactggaatgg
R1403A R	cccgggagtctcgctgccgctgcaaccgcctttggtcgggctcttcgggctgttgctgtt accggtaaacactttggtttcaccggtagcgccGGCcttcttcactgg
K1402A R1403A R	cccgggagtctcgctgccgctgcaaccgcctttggtcgggctcttcgggctgttgctgtt accggtaaacactttggtttcaccggtagcgccGGCCGCcactggaatgg
K1410A R	cccgggagtctcgctgccgctgcaaccgcctttggtcgggctcttcgggctgttgctgtt accggtaaacacGGCggtttcacc
K1420A R	cccgggagtctcgctgccgctgcaaccgcctttggtcgggctGGCcgggctgttgctg
K1424A R	cccgggagtctcgctgccgctgcaaccgccGGCggtcgggctcttcggg
E1347 F	ctatgccaatgccggtcatgtgGCCggtcagagcgccctgttcatg
E1347 R	catgaacagggcgctctgaccGGCcacatgaccggcattggcatag
error_prone PCR F	tgagccagctgggcggcgacagcggcagcgagactcccgggacctcagagtccgccacacc cgaaagtggcagctacgccctgggtcc
error_prone PCR R	aatagggccctctagatgcatgctcgagttagcaaccgcctttggtcggg
TALE Right DddA F	tgaagaaaggtctgggcggatccggcagctacgccctgggtccgtatcag
TALE_Left_DddA_F	tgaagaaaggcctgggtggatccggcagctacgccctgggtccgtatcag
TALE_DddA R	tcagattagttgagccgccagagcaaccgcctttggtcgggctcttcggg
TALE_UGI F	tctggcggctcaactaatctgagcgacatcattg
TALE_ UGI R	cagatccgaaaatggatatacaagctccc
mRNA F	gaaattaatacgactcactatagggagacccaagctggctagcaccgccaccatggccctgt cccgtgcggtttgtggcac
mRNA R	agggaagaaagcgaaaggag
CMV for AAV F	ggcctcagtgagcgagcgagcgcgcagagagggagtggccaactccatcactaggggttcctgcgg ccgcacgcgtcgatgtacgggccagatatacgcgttgacattg
CMV for AAV R	gagccatggtggcagcctgctttttttgtacaaacttgaatttcgataagccagtaagcagtgggttc
ND1 for AAV F	caaaaaagcaggctgccaccatggctcttagtcgagccgtctgtggtacttcccg
ND4 for AAV F	acgatgtccctgattatgctgggatccgaattcaagatctgcgtaccctgggttacag
ND1, 4 for AAV R	ctagcatttaggtgacactatagaatagggccctcacagcatcttgatcttattctcgc

Supplementary Table 2.

Name	sgRNA sequence 5` to 3`
HEK3	GGCCCAGACTGAGCACGTGATGG
TYRO3	GGCCACACTAGCGTTGCTGCTGG
ROR1 site1	GCCATAGATGGTGGACCGAAAGG
ROR1 site2	CCATCTATGGCTCTCGGCTGCGG
ROR1 site3	CCGCAGCCGAGAGCCATAGATGG
FANCF	GGAATCCCTTCTGCAGCACCTGG
HBB	CTTGCCCCACAGGGCAGTAACGG
EMX1 site 1	CCCTGGCCCAGGTGAAGGTGTGG
EMX1 site 2	AAAGTACAAACGGCAGAAGCTGG
TRAC5 site 1	GTGGTAGCGGAACTCACTAAGGGG
TRAC5 site 2	CACCCAGCCTGCTCTGCCTTGGG

Supplementary Table 3.

	Forward primer(5` to 3`)	Reverse primer(5` to 3`)
FANCF	ggagacgttcatgactggca	gggcctggaagttcgctaat
TYRO3	ctgtcaacaaagtgctggcc	agttacacagggccttcgtg
EMX1	tgccatccccttctgtgaat	ccattgcttgtccctctgtc
ROR1	ttccttcctgtccctgtgga	ctctcacctgcctcctctct
HBB	aacagcatcaggagtggaca	aagagccaaggacaggtacg
HEK3	cagggagcttggcatgagaa	gctccatcactttctggcct
TRAC5 site 1	caagcaacagtactcacataggc	cagattctgtgcattcttccag
TRAC5 site 2	ggacccgaggtattgtgatg	ttaggatgcacccagagacc
Hman ND1	ggttcggttggtctctgcta	atggccaacctcctactcct
Hman ND4	gccattctcatccaaacc	ggttgagggataggaggag
Hman ND6	ggtttgtggggttttcttct	caaccaccaccccatcata
mouse ND5	cgcagctacaggaaaatcagc	atggtattcctgtgagggcg
Hman MTND4P12	ctaattctctttgaggagcatggttag	tatcacttccagccacctatttcc

Supplementary Table 4.

	Sequence 5` to 3`
FANCF F	acactctttccctacacgacgctcttccgatcttcgcggatgttccaatcagt
FANCF R	gtgactggagttcagacgtgtgctcttccgatctgatggatgtggcgcaggtag
TYRO3 F	acactctttccctacacgacgctcttccgatctctgtcaacaaagtgctggcc
TYRO3 R	gtgactggagttcagacgtgtgctcttccgatctcttgactcccatgcctcctg
EMX1 F	acactctttccctacacgacgctcttccgatctagcctcagtcttcccatcag
EMX1 R	gtgactggagttcagacgtgtgctcttccgatctatcgatgtcctccccattgg
ROR1 F	acactctttccctacacgacgctcttccgatctcacctccttgccgtttgttg
ROR1 R	gtgactggagttcagacgtgtgctcttccgatctgcactgcaaagtctctggga
HBB F	acactctttccctacacgacgctcttccgatcttctccacatgcccagtttct
HBB R	gtgactggagttcagacgtgtgctcttccgatctgcataaaagtcagggcagag
HEK3 F	acactctttccctacacgacgctcttccgatctagacagggatcccagggaaa
HEK3 R	gtgactggagttcagacgtgtgctcttccgatctgagctgcacatactagcccc
TRAC5 site 1 F	acactctttccctacacgacgctcttccgaaatggtcctgtctctcaagaatcccc
TRAC5 site 1 R	gtgactggagttcagacgtgtgctcttccgatcttcttccagggccaacagtattgtg
TRAC5 site 2 F	acactctttccctacacgacgctcttccgatctctagtcggtgtttcctgtcttgaaacac
TRAC5 site 2 R	gtgactggagttcagacgtgtgctcttccgatctgtatctgtaaaaccaagaggccacagcgg
Hman ND1 F	acactctttccctacacgacgctcttccgatctaagggtggagaggttaaaggag
Hman ND1 R	gtgactggagttcagacgtgtgctcttccgatctccctggtcaacctcaaccta
Hman ND4 F	acactctttccctacacgacgctcttccgatctgacttcaaactctactcccactaatag
Hman ND4 R	gtgactggagttcagacgtgtgctcttccgatctgttgtggtaaatatgtagagggag
Hman ND6 F	acactctttccctacacgacgctcttccgatctctctttcacccacagcacc
Hman ND6 R	gtgactggagttcagacgtgtgctcttccgatctgattgttagcggtgtggtcg
mouse ND5 F	acactctttccctacacgacgctcttccgatctcctccacccatgactaccat
mouse ND5 R	gtgactggagttcagacgtgtgctcttccgatctgggtgagagcacaaatagctg
Hman MTND4P12 F	acactctttccctacacgacgctcttccgatctggctgagcgagcctca
Hman MTND4P12 R	gtgactggagttcagacgtgtgctcttccgatctgacttctagcaagcctcactaatc

Supplementary Table 5.

Forwad primer	adapter sequence	Sequence 5` to 3`
D501	tatagcct	AATGATACGGCGACCACCGAGATCTACACtatagcctACACTCTTTCCCTACACGAC
D502	atagaggc	AATGATACGGCGACCACCGAGATCTACACatagaggcACACTCTTTCCCTACACGAC
D503	cctatcct	AATGATACGGCGACCACCGAGATCTACACcctatcctACACTCTTTCCCTACACGAC
D504	ggctctga	AATGATACGGCGACCACCGAGATCTACACggctctgaACACTCTTTCCCTACACGAC
D505	aggcgaag	AATGATACGGCGACCACCGAGATCTACACaggcgaagACACTCTTTCCCTACACGAC
D506	taatctta	AATGATACGGCGACCACCGAGATCTACACtaatcttaACACTCTTTCCCTACACGAC
D507	caggacgt	AATGATACGGCGACCACCGAGATCTACACcaggacgtACACTCTTTCCCTACACGAC
D508	gtactgac	AATGATACGGCGACCACCGAGATCTACACgtactgacACACTCTTTCCCTACACGAC

Reverse primer		
D701	cgagtaat	CAAGCAGAAGACGGCATACGAGATcgagtaatGTGACTGGAGTTCAGACGTGT
D702	tctccgga	CAAGCAGAAGACGGCATACGAGATtctccggaGTGACTGGAGTTCAGACGTGT
D703	aatgagcg	CAAGCAGAAGACGGCATACGAGATaatgagcgGTGACTGGAGTTCAGACGTGT
D704	ggaatctc	CAAGCAGAAGACGGCATACGAGATggaatctcGTGACTGGAGTTCAGACGTGT
D705	ttctgaat	CAAGCAGAAGACGGCATACGAGATttctgaatGTGACTGGAGTTCAGACGTGT
D706	acgaattc	CAAGCAGAAGACGGCATACGAGATacgaattcGTGACTGGAGTTCAGACGTGT
D707	agcttcag	CAAGCAGAAGACGGCATACGAGATagcttcagGTGACTGGAGTTCAGACGTGT
D708	gcgcatta	CAAGCAGAAGACGGCATACGAGATgcgcattaGTGACTGGAGTTCAGACGTGT
D709	catagccg	CAAGCAGAAGACGGCATACGAGATcatagccgGTGACTGGAGTTCAGACGTGT
D710	ttcgcgga	CAAGCAGAAGACGGCATACGAGATttcgcggaGTGACTGGAGTTCAGACGTGT
D711	gcgcgaga	CAAGCAGAAGACGGCATACGAGATgcgcgagaGTGACTGGAGTTCAGACGTGT
D712	ctatcgct	CAAGCAGAAGACGGCATACGAGATctatcgctGTGACTGGAGTTCAGACGTGT

Supplementary Table 6.

Name	Sequence 5` to 3`
Mito 9397-1892 F	aaagcacataccaaggccac
Mito 9397-1892 R	ttggctctccttgcaaagtt
Mito 15195-9796 F	tatccgccatcccatacatt
Mito 15195-9796 R	aatgttgagccgtagatgcc
Mito 2478-10858 F	gcaaatcttaccccgcctg
Mito 2478-10858 R	aattaggctgtgggtggttg
Mito 10653-2688 F	gccatactagtctttgccgc
Mito 10653-2688 R	ggcaggtcaatttcactgg

Supplementary Sequence 1.

All DddAtox variants-Cas9 and Cas9 - DddAtox variants have the general architecture (from N to C-terminus): DddA variants-16aa linker- Cas9-4aa linker-UGI-4aa linker-SV40 NLS and SV40 NLS-4aa linker-UGI-4aa linker- Cas9-16aa linker DddAtox variants

SV40 NLS

PKKKRKVV

DddA AAAAA

GSYALGPYQISAPQLPAYNGQTVGTFYYVNDAGGLESKVFSSGGPTPYPNYANAGHVEGQSALFMR
DNGISEGLVFHNNPEGTCGFCVNMTETLLPENAKMTVVPPEGAIPVAAGATGETAVFTGNSNSPASPT
AGGC

E1347A

GSYALGPYQISAPQLPAYNGQTVGTFYYVNDAGGLESKVFSSGGPTPYPNYANAGHVAGQSALFMR
DNGISEGLVFHNNPEGTCGFCVNMTETLLPENAKMTVVPPEGAIPVKRGATGETKVFTGNSNSPKSP
TKGGC

GSVG

GSYALGPYQISAPQLPAYNGQTVGTFYYVNDAGGLEGKVFSSGGPTPYPNYANAGHVESQSALFMR
DNGISEGLVFHNNPEGTCGFCVNMTETLLPENAKMTVVPPEGVIPVKRGATGETKVFTGNSNGPKSP
TKGGC

4aa linker

SGGS

16aa Linker

SGSETPGTSESATPES

SpCas9

DKKYSIGLDIGTNSVGWAVITDEYKVPSKKFKVLGNTDRHSIKKNLIGALLFDSGETAEATRLKRTARRR
YTRRKNRICYLQEIFSNEMAKVDDSFFHRLEESFLVEEDKKHERHPIFGNIVDEVAYHEKYPTIYHLRKK
LVDSTDKADLRLIYLALAHMIKFRGHFLIEGDLNPDNSDVDKLFIQLVQTYNQLFEENPINASGVDAKAIL
SARLSKSRRLENLIAQLPGEKKNGLFGNLIALSLGLTPNFKSNFDLAEDAKLQLSKDTYDDDLDNLLAQ
IGDQYADLFLAAKNLSDAILLSDILRVNTEITKAPLSASMIKRYDEHHQDLTLLKALVRQQLPEKYKEIFF
DQSKNGYAGYIDGGASQEEFYKFIKPILEKMDGTEELLVKLNREDLLRKQRTFDNGSIPHQIHLGELHA
ILRRQEDFYPFLKDNREKIEKILTFRIPYYVGPLARGNSRFAWMTRKSEETITPWNFEEVVDKGASAQS
FIERMTNFDKNLPNEKVLPKHSLLYEYFTVYNELTKVKYVTEGMRKPAFLSGEQKKAIVDLLFKTNRKV
TVKQLKEDYFKKIECFDSVEISGVEDRFNASLGTYHDLLKIIKDKDFLDNEENEDILEDIVLTLTLFEDRE
MIEERLKTYAHLFDDKVMKQLKRRRYTGWGRLSRKLINGIRDKQSGKTILDFLKSDGFANRNFMQLIH
DDSLTFKEDIQKAQVSGQGDSLHEHIANLAGSPAIKKGILQTVKVVDELVKVMGRHKPENIVIEMAREN
QTTQKGQKNSRERMKRIEEGIKELGSQILKEHPVENTQLQNEKLYLYYLQNGRDMYVDQELDINRLSD
YDVDHIVPQSFLKDDSIDNKVLTRSDKNRGKSDNVPSEEVVKKMKNYWRQLLNAKLITQRKFDNLTKA

ERGGLSELDKAGFIKRQLVETRQITKHVAQILDSRMNTKYDENDKLIREVKVITLKSKLVSDFRKDFQFY
KVREINNYHHAHDAYLNAVVGTALIKKYPKLESEFVYGDYKVYDVRKMIAKSEQEIGKATAKYFFYSNI
MNFFKTEITLANGEIRKRPLIETNGETGEIVWDKGRDFATVRKVLSMPQVNIVKKTEVQTGGFSKESIL
PKRNSDKLIARKKDWDPKKYGGFDSPTVAYSVLVVAKVEKGKSKKLKSVKELLGITIMERSSFEKNPID
FLEAKGYKEVKKDLIIKLPKYSLFELENGRKRMLASAGELQKGNELALPSKYVNFLYLASHYEKLKGSP
EDNEQKQLFVEQHKHYLDEIIEQISEFSKRVILADANLDKVLSAYNKHRDKPIREQAENIIHLFTLTNLGA
PAAFKYFDTTIDRKRYTSTKEVLDATLIHQSITGLYETRIDLSQLGGD

SpnCas9(D10A)

DKKYSIGLAIGTNSVGWAVITDEYKVPSKKFKVLGNTDRHSIKKNLIGALLFDSGETAEATRLKRTARRR
YTRRKNRICYLQEIFSNEMAKVDDSFFHRLEESFLVEEDKKHERHPIFGNIVDEVAYHEKYPTIYHLRKK
LVDSTDKADLRLIYLALAHMIKFRGHFLIEGDLNPDNSDVDKLFIQLVQTYNQLFEENPINASGVDAKAIL
SARLSKSRRLENLIAQLPGEKKNGLFGNLIALSLGLTPNFKSNFDLAEDAKLQLSKDTYDDDLDNLLAQ
IGDQYADLFLAAKNLSDAILLSDILRVNTEITKAPLSASMIKRYDEHHQDLTLLKALVRQQLPEKYKEIFF
DQSKNGYAGYIDGGASQEEFYKFIKPILEKMDGTEELLVKLNREDLLRKQRTFDNGSIPHQIHLGELHA
ILRRQEDFYPFLKDNREKIEKILTFRIPYYVGPLARGNSRFAWMTRKSEETITPWNFEEVVDKGASAQS
FIERMTNFDKNLPNEKVLPKHSLLYEYFTVYNELTKVKYVTEGMRKPAFLSGEQKKAIVDLLFKTNRKV
TVKQLKEDYFKKIECFDSVEISGVEDRFNASLGTYHDLLKIIKDKDFLDNEENEDILEDIVLTLTLFEDRE
MIEERLKTYAHLFDDKVMKQLKRRRYTGWGRLSRKLINGIRDKQSGKTILDFLKSDGFANRNFMQLIH
DDSLTFKEDIQKAQVSGQGDSLHEHIANLAGSPAIKKGILQTVKVVDELVKVMGRHKPENIVIEMAREN
QTTQKGQKNSRERMKRIEEGIKELGSQILKEHPVENTQLQNEKLYLYYLQNGRDMYVDQELDINRLSD
YDVDHIVPQSFLKDDSIDNKVLTRSDKNRGKSDNVPSEEVVKKMKNYWRQLLNAKLITQRKFDNLTKA
ERGGLSELDKAGFIKRQLVETRQITKHVAQILDSRMNTKYDENDKLIREVKVITLKSKLVSDFRKDFQFY
KVREINNYHHAHDAYLNAVVGTALIKKYPKLESEFVYGDYKVYDVRKMIAKSEQEIGKATAKYFFYSNI
MNFFKTEITLANGEIRKRPLIETNGETGEIVWDKGRDFATVRKVLSMPQVNIVKKTEVQTGGFSKESIL
PKRNSDKLIARKKDWDPKKYGGFDSPTVAYSVLVVAKVEKGKSKKLKSVKELLGITIMERSSFEKNPID
FLEAKGYKEVKKDLIIKLPKYSLFELENGRKRMLASAGELQKGNELALPSKYVNFLYLASHYEKLKGSP
EDNEQKQLFVEQHKHYLDEIIEQISEFSKRVILADANLDKVLSAYNKHRDKPIREQAENIIHLFTLTNLGA
PAAFKYFDTTIDRKRYTSTKEVLDATLIHQSITGLYETRIDLSQLGGD

SpnCa9(H840A)

DKKYSIGLDIGTNSVGWAVITDEYKVPSKKFKVLGNTDRHSIKKNLIGALLFDSGETAEATRLKRTARRR
YTRRKNRICYLQEIFSNEMAKVDDSFFHRLEESFLVEEDKKHERHPIFGNIVDEVAYHEKYPTIYHLRKK
LVDSTDKADLRLIYLALAHMIKFRGHFLIEGDLNPDNSDVDKLFIQLVQTYNQLFEENPINASGVDAKAIL
SARLSKSRRLENLIAQLPGEKKNGLFGNLIALSLGLTPNFKSNFDLAEDAKLQLSKDTYDDDLDNLLAQ
IGDQYADLFLAAKNLSDAILLSDILRVNTEITKAPLSASMIKRYDEHHQDLTLLKALVRQQLPEKYKEIFF
DQSKNGYAGYIDGGASQEEFYKFIKPILEKMDGTEELLVKLNREDLLRKQRTFDNGSIPHQIHLGELHA
ILRRQEDFYPFLKDNREKIEKILTFRIPYYVGPLARGNSRFAWMTRKSEETITPWNFEEVVDKGASAQS
FIERMTNFDKNLPNEKVLPKHSLLYEYFTVYNELTKVKYVTEGMRKPAFLSGEQKKAIVDLLFKTNRKV
TVKQLKEDYFKKIECFDSVEISGVEDRFNASLGTYHDLLKIIKDKDFLDNEENEDILEDIVLTLTLFEDRE
MIEERLKTYAHLFDDKVMKQLKRRRYTGWGRLSRKLINGIRDKQSGKTILDFLKSDGFANRNFMQLIH
DDSLTFKEDIQKAQVSGQGDSLHEHIANLAGSPAIKKGILQTVKVVDELVKVMGRHKPENIVIEMAREN
QTTQKGQKNSRERMKRIEEGIKELGSQILKEHPVENTQLQNEKLYLYYLQNGRDMYVDQELDINRLSD
YDVDAIVPQSFLKDDSIDNKVLTRSDKNRGKSDNVPSEEVVKKMKNYWRQLLNAKLITQRKFDNLTKA
ERGGLSELDKAGFIKRQLVETRQITKHVAQILDSRMNTKYDENDKLIREVKVITLKSKLVSDFRKDFQFY
KVREINNYHHAHDAYLNAVVGTALIKKYPKLESEFVYGDYKVYDVRKMIAKSEQEIGKATAKYFFYSNI
MNFFKTEITLANGEIRKRPLIETNGETGEIVWDKGRDFATVRKVLSMPQVNIVKKTEVQTGGFSKESIL
PKRNSDKLIARKKDWDPKKYGGFDSPTVAYSVLVVAKVEKGKSKKLKSVKELLGITIMERSSFEKNPID
FLEAKGYKEVKKDLIIKLPKYSLFELENGRKRMLASAGELQKGNELALPSKYVNFLYLASHYEKLKGSP
EDNEQKQLFVEQHKHYLDEIIEQISEFSKRVILADANLDKVLSAYNKHRDKPIREQAENIIHLFTLTNLGA
PAAFKYFDTTIDRKRYTSTKEVLDATLIHQSITGLYETRIDLSQLGGD

SpdCas9(D10A, H840A)

DKKYSIGLAIGTNSVGWAVITDEYKVPSKKFKVLGNTDRHSIKKNLIGALLFDSGETAEATRLKRTARRR
YTRRKNRICYLQEIFSNEMAKVDDSFFHRLEESFLVEEDKKHERHPIFGNIVDEVAYHEKYPTIYHLRKK
LVDSTDKADLRLIYLALAHMIKFRGHFLIEGDLNPDNSDVDKLFIQLVQTYNQLFEENPINASGVDAKAIL
SARLSKSRRLENLIAQLPGEKKNGLFGNLIALSLGLTPNFKSNFDLAEDAKLQLSKDTYDDDLDNLLAQ
IGDQYADLFLAAKNLSDAILLSDILRVNTEITKAPLSASMIKRYDEHHQDLTLLKALVRQQLPEKYKEIFF
DQSKNGYAGYIDGGASQEEFYKFIKPILEKMDGTEELLVKLNREDLLRKQRTFDNGSIPHQIHLGELHA
ILRRQEDFYPFLKDNREKIEKILTFRIPYYVGPLARGNSRFAWMTRKSEETITPWNFEEVVDKGASAQS
FIERMTNFDKNLPNEKVLPKHSLLYEYFTVYNELTKVKYVTEGMRKPAFLSGEQKKAIVDLLFKTNRKV
TVKQLKEDYFKKIECFDSVEISGVEDRFNASLGTYHDLLKIIKDKDFLDNEENEDILEDIVLTLTLFEDRE
MIEERLKTYAHLFDDKVMKQLKRRRYTGWGRLSRKLINGIRDKQSGKTILDFLKSDGFANRNFMQLIH
DDSLTFKEDIQKAQVSGQGDSLHEHIANLAGSPAIKKGILQTVKVVDELVKVMGRHKPENIVIEMAREN
QTTQKGQKNSRERMKRIEEGIKELGSQILKEHPVENTQLQNEKLYLYYLQNGRDMYVDQELDINRLSD
YDVDAIVPQSFLKDDSIDNKVLTRSDKNRGKSDNVPSEEVVKKMKNYWRQLLNAKLITQRKFDNLTKA
ERGGLSELDKAGFIKRQLVETRQITKHVAQILDSRMNTKYDENDKLIREVKVITLKSKLVSDFRKDFQFY
KVREINNYHHAHDAYLNAVVGTALIKKYPKLESEFVYGDYKVYDVRKMIAKSEQEIGKATAKYFFYSNI
MNFFKTEITLANGEIRKRPLIETNGETGEIVWDKGRDFATVRKVLSMPQVNIVKKTEVQTGGFSKESIL
PKRNSDKLIARKKDWDPKKYGGFDSPTVAYSVLVVAKVEKGKSKKLKSVKELLGITIMERSSFEKNPID
FLEAKGYKEVKKDLIIKLPKYSLFELENGRKRMLASAGELQKGNELALPSKYVNFLYLASHYEKLKGSP
EDNEQKQLFVEQHKHYLDEIIEQISEFSKRVILADANLDKVLSAYNKHRDKPIREQAENIIHLFTLTNLGA
PAAFKYFDTTIDRKRYTSTKEVLDATLIHQSITGLYETRIDLSQLGGD

UGI

TNLSDIIEKETGKQLVIQESILMLPEEVEEVIGNKPESDILVHTAYDESTDENVMLLTSDAPEYKPWALVI
QDSNGENKIKML

Supplementary Sequence 2.

Left- SOD2 MTS-3XHA –N-terminal domain-TALE repeat-C-terminal domain- 2aa linker-DddA$_{tox}$ half or GSVG or E1347A-4aa linker-UGI

Right- COX8A MTS-3XFLAG –N-terminal domain-TALE repeat-C-terminal domain-2aa linker-DddA$_{tox}$ half or GSVG or E1347A-4aa linker-UGI

tRNA-Cys- COX8A MTS-3XFLAG –N-terminal domain-TALE repeat-C-terminal domain-2aa linker-GSVG or E1347A-4aa linker-UGI

E1347A

GSYALGPYQISAPQLPAYNGQTVGTFYYVNDAGGLESKVFSSGGPTPYPNYANAGHVAGQSALFMR
DNGISEGLVFHNNPEGTCGFCVNMTETLLPENAKMTVVPPEGAIPVKRGATGETKVFTGNSNSPKSP
TKGGC

GSVG

GSYALGPYQISAPQLPAYNGQTVGTFYYVNDAGGLEGKVFSSGGPTPYPNYANAGHVESQSALFMR
DNGISEGLVFHNNPEGTCGFCVNMTETLLPENAKMTVVPPEGVIPVKRGATGETKVFTGNSNGPKSP
TKGGC

1333N

GSYALGPYQISAPQLPAYNGQTVGTFYYVNDAGGLESKVFSSGG

1333C

PTPYPNYANAGHVEGQSALFMRDNGISEGLVFHNNPEGTCGFCVNMTETLLPENAKMTVVPPEGAIP
VKRGATGETKVFTGNSNSPKSPTKGGC

1397N

GSYALGPYQISAPQLPAYNGQTVGTFYYVNDAGGLESKVFSSGGPTPYPNYANAGHVEGQSALFMR
DNGISEGLVFHNNPEGTCGFCVNMTETLLPENAKMTVVPPEG

1397C

AIPVKRGATGETKVFTGNSNSPKSPTKGGC

hND1 Left

MALSRAVCGTSRQLAPVLGYLGSRQKHSLPDYPYDVPDYAGYPYDVPDYAGYPYDVPDYAMDIADL
RTLGYSQQQQEKIKPKVRSTVAQHHEALVGHGFTHAHIVALSQHPAALGTVAVKYQDMIAALPEATHE
AIVGVGKQWSGARALEALLTVAGELRGPPLQLDTGQLLKIAKRGGVTAVEAVHAWRNALTGAPLNLTP
DQVVAIAS**HD**GGKQALETVQRLLPVLCQAHGLLTPDQVVAIAS**NG**GGKQALETVQRLLPVLCQDHGL
TPAQVVAIAS**NI**GGKQALETVQRLLPVLCQDHGLTPDQVVAIAS**NN**GGKQALETVQRLLPVLCQAHGL
TPDQVVAIAS**HD**GGKQALETVQRLLPVLCQDHGLTPDQVVAIAS**HD**GGKQALETVQRLLPVLCQAHG
LTPAQVVAIAS**NG**GGKQALETVQRLLPVLCQDHGLTPAQVVAIAS**NI**GGKQALETVQRLLPVLCQAHGL
TPDQVVAIAS**NN**GGKQALETVQRLLPVLCQAHGLTPAQVVAIAS**HD**GGKQALETVQRLLPVLCQDHG
LTPDQVVAIAS**HD**GGKQALETVQRLLPVLCQAHGLTPEQVVAIAS**NN**GGKQALETVQRLLPVLCQAHG

LTPAQVVAIAS**NG**GGKQALETVQRLLPVLCQAHGLTPAQVVAIAS**NG**GGKQALETVQRLLPVLCQAHG
TPEQVVAIASNGGGKQALESIVAQLSRPDPALAALTNDHLVALACLGGRPALDAVKKGLG

hND1 Right

MASVLTPLLLRGLTGSARRLPVPRAKIHSLDYKDHDGDYKDHDIDYKDDDDKAMDIADLRTLGYSQQQ
QEKIKPKVRSTVAQHHEALVGHGFTHAHIVALSQHPAALGTVAVKYQDMIAALPEATHEAIVGVGKQW
SGARALEALLTVAGELRGPPLQLDTGQLLKIAKRGGVTAVEAVHAWRNALTGAPLNLTPDQVVAIAS**NN**
GGKQALETVQRLLPVLCQDHGLTPAQVVAIAS**NI**GGKQALETVQRLLPVLCQAHGLTPEQVVAIAS**NN**
GGKQALETVQRLLPVLCQAHGLTPDQVVAIAS**NG**GGKQALETVQRLLPVLCQAHGLTPAQVVAIAS**N**
GGGKQALETVQRLLPVLCQAHGLTPEQVVAIAS**NG**GGKQALETVQRLLPVLCQAHGLTPAQVVAIAS**N**
NGGKQALETVQRLLPVLCQDHGLTPAQVVAIAS**NI**GGKQALETVQRLLPVLCQAHGLTPEQVVAIAS**N**
GGGKQALETVQRLLPVLCQAHGLTPDQVVAIAS**NN**GGKQALETVQRLLPVLCQAHGLTPAQVVAIAS**H**
DGGKQALETVQRLLPVLCQDHGLTPDQVVAIAS**NG**GGKQALETVQRLLPVLCQAHGLTPDQVVAIAS
HDGGKQALETVQRLLPVLCQAHGLTPDQVVAIAS**NI**GGKQALETVQRLLPVLCQAHGLTPAQVVAIAS
HDGGKQALETVQRLLPVLCQAHGLTPAQVVAIAS**HD**GGKQALETVQRLLPVLCQDHGLTPAQVVAIAS
HDGGKQALETVQRLLPVLCQAHGTPEQVVAIASNGGGKQALESIVAQLSRPDPALAALTNDHLVALAC
LGGRPALDAVKKGLG

mND5 Left

MALSRAVCGTSRQLAPVLGYLGSRQKHSLPDYPYDVPDYAGYPYDVPDYAGYPYDVPDYAGIRIQDL
RTLGYSQQQQEKIKPKVRSTVAQHHEALVGHGFTHAHIVALSQHPAALGTVAVKYQDMIAALPEATHE
AIVGVGKQWSGARALEALLTVAGELRGPPLQLDTGQLLKIAKRGGVTAVEAVHAWRNALTGAPLNLTP
DQVVAIAS**NG**GGKQALETVQRLLPVLCQAHGLTPAQVVAIAS**NG**GGKQALETVQRLLPVLCQAHGLT
PEQVVAIAS**NG**GGKQALETVQRLLPVLCQAHGLTPDQVVAIAS**HD**GGKQALETVQRLLPVLCQAHGL
TPAQVVAIAS**HD**GGKQALETVQRLLPVLCQDHGLTPAQVVAIAS**NG**GGKQALETVQRLLPVLCQDHG
LTPEQVVAIAS**NI**GGKQALETVQRLLPVLCQAHGLTPDQVVAIAS**HD**GGKQALETVQRLLPVLCQAHG
LTPDQVVAIAS**NG**GGKQALETVQRLLPVLCQAHGLTPAQVVAIASNNGGKQALETVQRLLPVLCQDH
GLTPAQVVAIAS**NN**GGKQALETVQRLLPVLCQDHGLTPDQVVAIAS**NG**GGKQALETVQRLLPVLCQA
HGLTPAQVVAIAS**HD**GGKQALETVQRLLPVLCQDHGLTPDQVVAIAS**HD**GGKQALETVQRLLPVLCQA
HGLTPEQVVAIAS**NN**GGKQALETVQRLLPVLCQAHGLTPEQVVAIAS**NI**GGKQALETVQRLLPVLCQA
HGLTPDQVVAIAS**NG**GGKQALETVQRLLPVLCQDHGTPEQVVAIASNGGGKQALESIVAQLSRPDPAL
AALTNDHLVALACLGGRPALDAVKKGLG

mND5 Right

MASVLTPLLLRGLTGSARRLPVPRAKIHSLDYKDHDGDYKDHDIDYKDDDDKAGIRIQDLRTLGYSQQ
QQEKIKPKVRSTVAQHHEALVGHGFTHAHIVALSQHPAALGTVAVKYQDMIAALPEATHEAIVGVGKQ
WSGARALEALLTVAGELRGPPLQLDTGQLLKIAKRGGVTAVEAVHAWRNALTGAPLNLTPEQVVAIAS
NGGGKQALETVQRLLPVLCQAHGLTPAQVVAIAS**NI**GGKQALETVQRLLPVLCQDHGLTPAQVVAIAS
NIGGKQALETVQRLLPVLCQAHGLTPDQVVAIAS**NI**GGKQALETVQRLLPVLCQAHGLTPDQVVAIAS**N**
IGGKQALETVQRLLPVLCQDHGLTPDQVVAIAS**NG**GGKQALETVQRLLPVLCQAHGLTPDQVVAIAS**NI**
GGKQALETVQRLLPVLCQAHGLTPAQVVAIAS**NI**GGKQALETVQRLLPVLCQDHGLTPAQVVAIAS**NI**G
GKQALETVQRLLPVLCQAHGLTPAQVVAIAS**NN**GGKQALETVQRLLPVLCQDHGLTPEQVVAIAS**NG**G
GKQALETVQRLLPVLCQAHGLTPAQVVAIAS**NG**GGKQALETVQRLLPVLCQDHGLTPAQVVAIAS**NI**G
GKQALETVQRLLPVLCQAHGLTPDQVVAIAS**NG**GGKQALETVQRLLPVLCQAHGLTPDQVVAIAS**NG**
GGKQALETVQRLLPVLCQAHGLTPEQVVAIAS**NI**GGKQALETVQRLLPVLCQAHGLTPDQVVAIAS**NG**
GGKQALETVQRLLPVLCQDHGTPEQVVAIASNGGGKQALESIVAQLSRPDPALAALTNDHLVALACLG
GRPALDAVKKGLG

tRNA-Cys

MASVLTPLLLRGLTGSARRLPVPRAKIHSLDYKDHDGDYKDHDIDYKDDDDKAGIRIQDLRTLGYSQQ
QQEKIKPKVRSTVAQHHEALVGHGFTHAHIVALSQHPAALGTVAVKYQDMIAALPEATHEAIVGVGKQ

WSGARALEALLTVAGELRGPPLQLDTGQLLKIAKRGGVTAVEAVHAWRNALTGAPLNLTPAQVVAIAS
NGGGKQALETVQRLLPVLCQDHGLTPEQVVAIAS**HD**GGKQALETVQRLLPVLCQAHGLTPAQVVAIAS
NIGGKQALETVQRLLPVLCQDHGLTPDQVVAIAS**NG**GGKQALETVQRLLPVLCQAHGLTPDQVVAIAS
NIGGKQALETVQRLLPVLCQDHGLTPDQVVAIAS**NG**GGKQALETVQRLLPVLCQAHGLTPAQVVAIAS
NGGGKQALETVQRLLPVLCQAHGLTPAQVVAIAS**NN**GGKQALETVQRLLPVLCQDHGLTPAQVVAIAS
NIGGKQALETVQRLLPVLCQAHGLTPAQVVAIAS**NI**GGKQALETVQRLLPVLCQAHGLTPDQVVAIAS**N**
GGGKQALETVQRLLPVLCQAHGLTPDQVVAIAS**NG**GGKQALETVQRLLPVLCQAHGLTPAQVVAIAS
NNGGKQALETVQRLLPVLCQAHGLTPDQVVAIAS**HD**GGKQALETVQRLLPVLCQDHGLTPAQVVAIAS
NIGGKQALETVQRLLPVLCQAHGLTPDQVVAIAS**NI**GGKQALETVQRLLPVLCQAHGLTPDQVVAIAS**N**
IGGKQALETVQRLLPVLCQDHGLTPDQVVAIAS**NG**GGKQALETVQRLLPVLCQAHGTPEQVVAIASNG
GGKQALESIVAQLSRPDPALAALTNDHLVALACLGGRPALDAVKKGLG

2aa linker

GS

4aa linker

SGGS

UGI

TNLSDIIEKETGKQLVIQESILMLPEEVEEVIGNKPESDILVHTAYDESTDENVMLLTSDAPEYKPWALVI
QDSNGENKIKML

Supplementary Source data

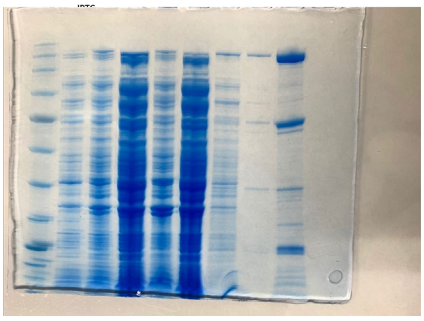

Supplement: Supplementary file 1 — Supplementary Information [file 41467_2022_31745_MOESM1_ESM.pdf]
