# Supplementary material for: Base editing in human cells with monomeric DddA-TALE fusion deaminases
Source: Nat Commun. 2022 Jul 12;13:4038. doi: 10.1038/s41467-022-31745-y (PMC9276701; doi:10.1038/s41467-022-31745-y)
Supplement: Supplementary file 3 — Description of Additional Supplementary Files [file 41467_2022_31745_MOESM3_ESM.pdf]

Description of Additional Supplementary Files

File Name: Supplementary Data 1
Description: In silico analysis of base-editable TC motifs in human mitochondrial DNA. For this analysis, reference sequences from the human mitochondrial genome (NC 012920) were used. The TALE array binds to DNA sequences represented by 5' binding seq and 3' binding seq, respectively. The length of the left and right TALE arrays is represented by L len and R len, respectively. S len represents the length of spacing regions
